# Gene mutations in a Han Chinese Alzheimer's disease cohort

**DOI:** 10.1002/brb3.1180

**Published:** 2018-12-14

**Authors:** Limin Ma, Jiewen Zhang, Yingying Shi, Wan Wang, Zhixia Ren, Mingrong Xia, Yuanxing Zhang, Miaomiao Yang

**Affiliations:** ^1^ Department of Neurology People's Hospital of Zhengzhou University Zhengzhou China; ^2^ Department of Neurology Henan Provincial People's Hospital Zhengzhou China; ^3^ Department of Neurology Xinxiang Medical University Xinxiang China

**Keywords:** Alzheimer's disease, frontotemporal dementia, gene mutation, *MAPT*, *PSEN1*, *PSEN2*, *TBK1*

## Abstract

**Objective:**

Alzheimer's disease (AD) is the most common form of dementia characterized by memory loss at disease onset. The gene mutations in the amyloid precursor protein (*APP*), presenilin 1 (*PSEN1*), and presenilin 2 (*PSEN2*) are the frequent causes of AD. However, the clinical and genetic features of AD overlap with other neurodegenerative diseases. The present study aimed to identify the clinical and genetic characteristics in a Han Chinese AD cohort.

**Methods:**

Detailed clinical assessment was applied to all the patients. We screened amyloid precursor protein (*APP*), *PSEN1*, *PSEN2*, and microtubule‐associated protein tau (*MAPT*) genes were assessed in 83 sporadic AD patients by Sanger sequencing. A total of 25 probands from families with AD were subjected to next‐generation sequencing on 53 dementia‐associated genes to capture the target region, and Sanger sequencing was used to detect the variants in the DNA sequence.

**Results:**

*PSEN1* p.L226R was found in an early‐onset AD (EOAD) family characterized by language impairment at disease onset, a novel probably pathogenetic variant (p.D534H) was identified in a frontal‐temporal dementia gene, TANK‐binding kinase 1 (*TBK1*) with a typical AD phenotype in a late‐onset AD (LOAD) family, and a *PSEN2*p.H169N mutation and two benign *MAPT* (p.Q230R and p.V48L) mutations were detected in three EOAD patients.

**Conclusions:**

Thus, five variants were identified in a Han Chinese cohort. In the present study, a novel, probably damaging FTLD gene *TBK1*variant with a typical AD phenotype was detected. Also, the phenotypic characteristics of* PSEN1* p.L226R, a *PSEN2*pathogenic mutation, and two likely benign *MAPT* variants were described. Hence, screening for mutations in other dementia genes could be further explored in clinically diagnosed AD patients.

## 1 INTRODUCTION

Dementia is a progressive neurodegenerative syndrome characterized by cognitive, behavioral, and neuropsychiatric changes that impair social function and activities of daily living (ADLs; Landeiro et al., 2018). Alzheimer's disease (AD) is the most common cause of dementia, and the pathological hallmarks include the presence of amyloid beta (Aβ) peptides and neurofibrillary tangles (Bagyinszky, Park, et al., 2016; Yagi et al., 2014), which account for 60%–80% of all the dementia cases (Che et al., 2018). According to the age of disease onset, the AD is classified into two types: early‐onset and late‐onset (Giri, Zhang, & Lu, 2016). The early‐onset AD (EOAD) refers to the age of disease onset <65 years, encompassing about 4%–6% of all the AD cases (Mendez, 2017). On the other hand, late‐onset AD (LOAD), which is the most common form, occurs in individuals >65 years of age (Sutovsky et al., 2018). Generally, AD is considered to be a polygenic disease resulting from complex interactions between environmental factors and several genes (Che et al., 2018). Reportedly, the three common causative genes associated with AD include the amyloid precursor protein (*APP*), presenilin 1 (*PSEN1*), and presenilin 2 (*PSEN2*) genes (Sutovsky et al., 2018). However, some rare frontotemporal lobar degeneration (FTLD) mutations in genes such as progranulin (*GRN*), *C9orf72*, and microtubule‐associated protein tau (*MAPT*) have also been described in clinical AD cohorts or families with AD clinical phenotypes (Piccoli et al., 2016).

Furthermore, the clinical features of AD are similar to that of other neurodegenerative diseases, such as FTLD and Parkinson's disease (PD), which might easily lead to misdiagnosis (Piccoli et al., 2016). In the present study, due to the clinical and genetic diversity of AD patients, we screened *PSEN1*, *PSEN2*, *MAPT*, and *APP* genes in 83 sporadic AD patients, while 53 dementia‐related genes which were screened based on previous studies (He et al., 2017; Hooli et al., 2014; Pottier et al., 2016; Vardarajan et al., 2015) and databases such as OMIM (http://www.omim.org/), Orphanet (http://www.orpha.net/consor/cgi-bin/index.php?lng=EN), and HGMD (http://www.hgmd.cf.ac.uk/ac/index.php) in 25 AD patients with a dementia family history. Consequently, two known mutations, a *PSEN1* p.L226R (chr14:73659480) mutation in an EOAD family and a *PSEN2* p.H169N (chr1:227075798) in a LOAD family, a novel FTLD gene TANK‐binding kinase 1 (*TBK1*) p.D534H (chr12:64889341) variant with typical AD phenotype in a LOAD family, and two probably benign *MAPT*variants p.Q230R and p.V48L (chr17:44060859 and chr17:44049233) in two sporadic EOAD patients, and some other insignificant variants were identified (online Supporting Information Table S2).

## 2 MATERIALS AND METHODS

### 2.1 Subjects

The study cohort consisted of 108 AD patients (83 sporadic AD patients and 25 probands from families with AD). All patients were enrolled from the Department of Neurology of People's Hospital of Zhengzhou University, evaluated, and clinically diagnosed as AD according to the criteria of the National Institute of Neurological and Communicative Disorders and Stroke‐Alzheimer's Disease and Related Disorders Association (NINCDS‐ADRDA; McKhann et al., 1984). The AD pedigree was defined as having at least one first‐ or second‐degree relative affected with dementia. In addition, blood samples were collected from eight family members of patient 1 (III‐1, III‐2, III‐3, III‐4, III‐5, III‐6, III‐7, and III‐8) and five family members of patient 4 (II‐1, II‐2, II‐3, III‐1, and III‐2). A total of 100 unrelated normal subjects were also recruited. The study protocol was approved by the Institutional Review Board of People's Hospital of Zhengzhou University.

### 2.2 Clinical and imaging assessment of patients

The medical and family history was obtained from the patient's caregivers or relatives. The patients underwent magnetic resonance imaging (MRI), magnetic resonance angiography (MRA) and/or 18F‐fludeoxyglucose positron emission tomography (18F‐FDG‐PET), and computed tomographic angiography (CTA). The neuropsychological assessment such as clinical dementia rating (CDR), mini‐mental state examination (MMSE), and ADL was applied. All patients underwent laboratory evaluations such as blood cell count, the levels of thyroid hormone, erythrocyte sedimentation rate, C‐reactive protein, folic acid, and vitamin B12.

### 2.3 Mutation screening and analysis

Blood samples from all patients and 100 unrelated normal subjects were collected in 5 ml EDTA anticoagulant tubes after informed consent was obtained from each participant. Genomic DNA was extracted from peripheral blood leukocytes using standard procedures. The coding exons, splice sites, and the adjacent intron sequences were analyzed for four genes (*PSEN1*, *PSEN2*, *APP*, and *MAPT*) in sporadic patients. Subsequently, these coding exons, splice sites, the immediately adjacent intron sequences, and high‐throughput sequencing data were analyzed in 53 genes associated with dementia in patients with a family history in order to identify the target region (Supporting Information Table S1). Briefly, point mutations and insertions/deletions were designed for target genes encompassing all exons and the splicing sites immediately adjacent to the intron sequences. The mutations in the target genes were screened by next‐generation sequencing (NGS) (Illumina HiSeq 2500, USA) with ≥200‐fold average high‐throughput sequencing depth; subsequently, the mutations were analyzed by bioinformatics. Sanger sequencing was used to detect the DNA sequence variants in the patients. The 100 unrelated normal subjects were screened only for *MAPT* and *TBK1* genes. The target site primers were designed based on the target gene mutation, and PCR amplification was performed using MultiGene OptiMax (Labnet, USA) under the following conditions: 94°C for 30 s, 55°C for 30 s, and 72°C for 1 min. Then, the PCR products of the proband and three other family members were purified and directly sequenced on an ABI3100 automated sequencer (Applied Biosystems, Foster City, CA, USA). The sequencing reads and lists were compared using Chromas software and NCBI blast. The single nucleotide polymorphisms were determined using the HGMD (http://www.hgmd.org; RRID:SCR_001888), NCBI dbSNP137 (RRID:SCR_002338, http://scicrunch.org/resolver/SCR_002338), Molgen (http://www.molgen.ua.ac.be/ADmuattions/, RRID:SCR_005700), HapMap (http://www.hapmap.org/index.html.en, RRID:SCR_002846), 1,000 human genome data set (http://www.1000genomes.org/, http://scicrunch.org/resolver/SCR_006828), and ExAC Browser Beta (http://exac.broadinstitute.org/, RRID:SCR_004068). The pathogenicity of the novel single nucleotide mutation was predicted using PolyPhen‐2 (http://genetics.bwh.harvard.edu/pph2/, RRID:SCR_016630) and SIFT (http://sift.jcvi.org/, RRID:SCR_012813) along with the conservation score.

## 3 RESULTS

### 3.1 Gene analysis

**Figure 1:**
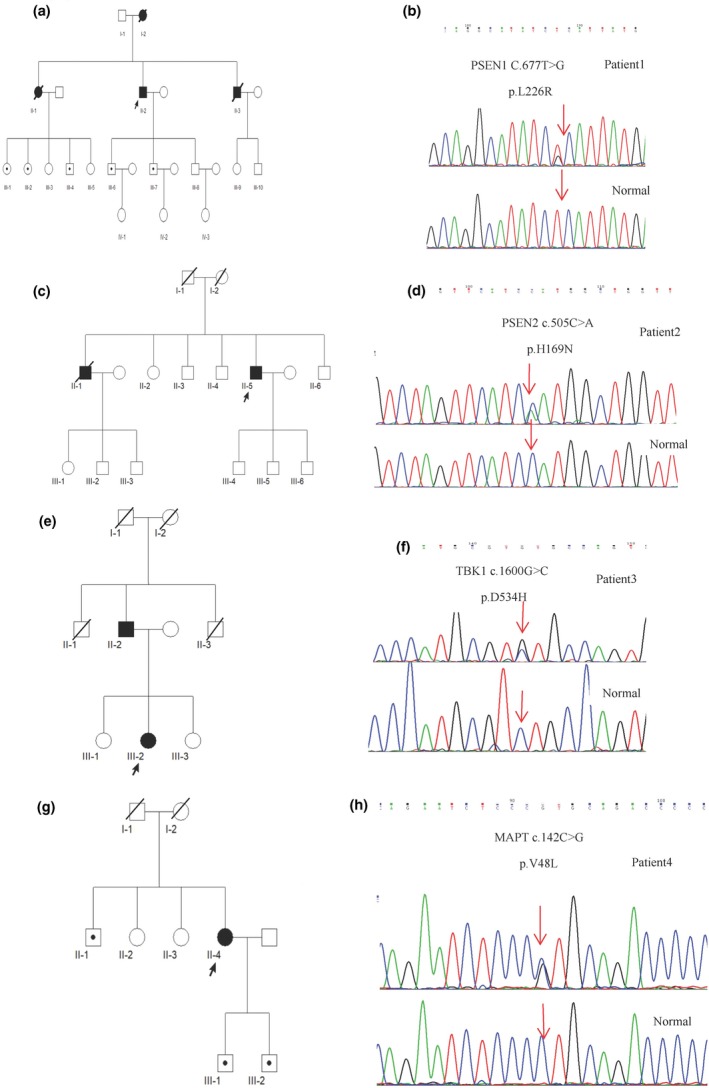
(a) The family tree of the PSEN1 p.L226R mutation pedigree. The arrow indicates the proband (II‐2); (b) DNA sequencing chromatograph of exon 7 of the PSEN1 gene (abnormal and normal), PSEN1 gene with a heterozygous mutation: C.677T>G p.L226R. (c) The family tree of the PSEN2 p.H169N mutation pedigree. The arrow indicates the proband (II‐5); (d) DNA sequencing chromatograph of exon 4 of the PSEN2 gene (abnormal and normal), PSEN2 gene with a heterozygous mutation: c.505C>A p.H169N. (e) The family tree of the TBK1 p.D534H mutation pedigree. The arrow indicates the proband (III‐2); (f) DNA sequencing chromatograph of exon 14 of the TBK1 gene (abnormal and normal), TBK1 gene with a heterozygous mutation: c.1600G>C p.D534H. (g) The family tree of the *MAPT* p.V48L mutation. The arrow indicates the proband (II‐4); (h) DNA sequencing chromatograph of exon 3 of the *MAPT* gene (abnormal and normal), *MAPT* gene with a heterozygous mutation: c.142C>G p.V48L. Square: male; circle: female; black symbol: affected family member; blackspot: carrier. The arrow indicates the mutation

A total of 108 AD patients, including 83 sporadic AD patients and 25 probands from families with AD, were included in this study. Interestingly, five heterozygous mutations were identified in four genes (Table 1), of which, two were known variants. One gene variant was *PSEN1* (c.677T>G p.L226R) mutation in an EOAD family (Figure 1a,b) with nine family members carrying the rare variant, while six members (II‐2, III‐1, III‐2, III‐4, III‐6, and III‐7) carried the variant (Figure 1a). The other variant was *PSEN2*(c.505C>A p.H169N) mutation in patient 2, with a LOAD family history (Figure 1c,d).

**Table 1:** The clinical and gene features of the variants

Patient	Age of onset (years)	Gene	Mutation	Family history	ExAC *N* (frequency)	First symptom	Neuropsychological assessment	Imaging features
1	60	PSEN1	c.677T>G p.L226R	Yes	—	Language impairment	CDR 2，MMSE 2/30, ADL 58/80	Brain CT images showed bilateral temporal lobe parenchyma and hippocampal atrophy. 18F‐FDG‐PET showed frontotemporal regions, parietal regions, hippocampal areas hypometabolism
2	63	PSEN2	c.505C>A p.H169N	Yes	0.0002	Memory loss	CDR 0.5, MMSE 23, ADL 20/80	Brain MRI images showed temporal areas, bilateral hippocampal atrophy (R > L)
3	68	TBK1	c.1600G>Cp.D534H	Yes	—	Memory loss	CDR1, MMSE22/30, ADL 27/80	Brain MRI showed bilateral hippocampal areas atrophy

Moreover, three novel heterozygous missense mutations were detected in this study. The novel *TBK1* (c.1600G>C p.D534H) variation in a LOAD family (Figure 1e,f) was not reported in The Human Genetics Mutation Database (HGMD), Molgen database, and previous literature. In addition, the mutation was not a single nucleotide polymorphism. The mutational analysis using SIFT and Polyphen‐2 predicted tolerated with a score of 0.121 and probably damaging with a score of 0.999, respectively. Two *MAPT* variants c.142C>G p.V48L and c.689A>G p.Q230R were detected in two sporadic cases. The other five family members of the *MAPT* p.V48L proband also harbored the rare variant of *MAPT* p.V48, while the II‐1, III‐1, and III‐2 members also carried the mutation (Figure 1g). Strikingly, no missense variation was found in the 100 unrelated normal controls screened for the three novel mutations.

### 3.2 Clinical features

The *PSEN1* p.L226R mutation was identified in patient 1, a 66‐year‐old man, who presented progressive discontinuous speech and stuttering at the age of 60 years. About after 3 years, his personality changed and was characterized by irritability. The condition deteriorated progressively accompanied by memory loss, stereotyped behavior, repeated questioning, paranoid thoughts, restlessness, violent behavior, forgetting names, prosopagnosia, and emotional vulnerability. Five members were affected in this family (Figure 1a), whose clinical features were similar to that of patient 1.

*PSEN2* p.H169N was detected in patient 2, a 63‐year‐old man with memory loss for 2 years and personality changes characterized by mild laziness, dependence on others, excessive sleep, and short temper for 1 year. His brother had irritability, bad temper, memory loss, prosopagnosia since 67 years of age, and died at the age of 72 years. The patient's mother died of tuberculosis at 57 years of age, and his father died at the age of 102 years without dementia. The family tree is illustrated in Figure 1c.

Patient 3 (*TBK1* p.D534H) was a 70‐year‐old woman suffering with memory loss and disorientation for about 2 years. Her father had unclassified dementia at 70 years of age and died in his 80s, while her uncles died young (Figure 1e).

### 3.3 Imaging features

Summarized in Table 1.

### 3.4 Neuropsychological assessment

Summarized in Table 1.

### 3.5 Auxiliary examinations

Intriguingly, no abnormalities were detected in laboratory examinations including blood cell count, thyroid hormone, erythrocyte sedimentation rate, C‐reactive protein, folic acid, and vitamin B12 in either of the patients.

## 4 DISCUSSION

Clinical and genetic crosstalk exists between AD and other neurodegenerative diseases, such as FTLD and PD (Piccoli et al., 2016); for example, AD with FTLD‐like phenotype (Zekanowski et al., 2006) and AD accompanied by myoclonus, seizures, and spastic paraparesis during the disease (Shea et al., 2016). The mutations in FTLD genes, *GRN*, *C9ORF72*, and *MAPT*, have been described in clinical AD cohorts or in pedigrees with clinical phenotypes of AD (Brouwers et al., 2007; Cacace et al., 2013; Rademakers et al., 2003). Thus, we screened all the dementia genes in our cohort with a dementia family history and identified two known pathological mutations (*PSEN1* p.L226R, *PSEN2* p.H169N), one novel heterozygous missense mutation in the FTLD gene with AD phenotype (TBK1 p.D534H), and two likely benign *MAPT* (p.Q230R, p.V48L) variants in a Han Chinese AD cohort.

The *PSEN1* p.L226R is localized in transmembrane domain 5 exon 7 of *PSEN1*protein and corresponds to a conserved amino acid residue in *PSEN2*(p.L232). In the current study, the *PSEN1* family demonstrated the clinical manifestation of language disability and altered personality at the beginning of the disease, which differed from the clinical manifestation of the initial memory loss in the AD. In addition, the *PSEN1*p.L226R mutation (Coleman, Kurlan, Crook, Werner, & Hardy, 2004) exhibited the clinical phenotype the proband, which was uncertain due to congenital retardation and personal bad habits. Thus, the *PSEN1* p.L226R variant family in thus study enriched the clinical manifestations of the *PSEN1* p.L226R variant worldwide. Moreover, *PSEN1* p.L226R encoded by codon 226 has been reported four times previously (Bagyinszky, Park, et al., 2016; Bagyinszky, Youn, An, & Kim, 2016; Coleman et al., 2004; Gomez‐Tortosa et al., 2010; Zekanowski et al., 2006), while p.L226F has been reported three times. The other three p.L226F families showed an early‐onset (33–37 years), AD or FTD‐like symptoms, and biopsy‐proved AD (Bagyinszky, Park, et al., 2016; Bagyinszky, Youn, et al., 2016). As compared to the reported L226F families, the p.L226R family in the current study had a later age of onset and similar language impairment as the first symptom; however, no Parkinsonism‐like syndrome was observed during the disease. These similar symptoms might be attributed to the L226F and L226R mutations that are amino acid substitutions resulting in substantial changes on the surface of the transmembrane domain of *PSEN1*(Zekanowski et al., 2006). The clinical differences could be ascribed to different ethnicities and environmental factors. However, compared to the other *PSEN1* variants in Han Chinese families (Deng et al., 2014; Dong et al., 2017; Jiang et al., 2015; Zhan et al., 2017), the cohort in this study was characterized by language disability and personality change converse to the initial memory loss.

*PSEN2*is a relatively rare mutation associated with dementia due to the late age of onset (Xia et al., 2015); while the mean age of onset could be 53.7 ± 7.8 years (Jayadev et al., 2010), some may be as late as the 70s (Xia et al., 2015). The *PSEN2* p.H169N is localized in the highly conserved domain in the protein, and the homologous amino acid residue *PSEN1* p.H175 has been reported previously. In the present study, the* PSEN2* p.H169N family showed the age of onset at 61 and 68 years, which was consistent with that described in the literature. In addition to patient 2, *PSEN2*p.H169N was detected in three patients (Shi et al., 2015), including two with AD and one with FTD. Strikingly, the three patients were Chinese, which might be ascribed to the large dementia population in China.

We found two rare *MAPT* (p.Q230R in exon 6 and p.V48L in exon 3) variations, which might be benign polymorphisms. As described previously, *MAPT* gene consists of 16 exons and expresses six isoforms in the human brain by alternative mRNA splicing of exons 2, 3, 4A, 6, 8, and 10 (Goedert & Spillantini, 2011; Park, Ahn, & Gallo, 2016), of which exons 6 and 8 are not expressed in any major brain isoforms (Rademakers, Cruts, & Broeckhoven, 2004). Interestingly, the exon 3 is spliced only when exon 2 is present (Park et al., 2016; Sergeant, Delacourte, & Buee, 2005); however, the exons 3 and 2 isoforms are expressed minimally (Park et al., 2016). In the current study, the p.V48 rare variant of *MAPT*was detected in the family, and the patient's brother harboring the same mutation is currently 62 years old, with normal cognition, thereby suggesting that p.V48L is putatively benign.

*TBK1* is a multifunctional kinase involved in the regulation of various cellular pathways, including immune response, inflammation, autophagy, cell proliferation, and insulin signaling (Freischmidt, Muller, Ludolph, Weishaupt, & Andersen, 2017). The loss of function of *TBK1*variants or functional deficits of *TBK1* missense mutations cause a dominant form of ALS, FTD, ALS‐FTD (Freischmidt et al., 2015), and other rare phenotypes such as corticobasal syndrome (CBS; van der Zee et al., 2017) and AD (Verheijen et al., 2018). The patient carrying *TBK1*p.D534H experienced memory loss and disorientation, which was in line with the diagnosis of AD. Thus, screening for mutations in *TBK1*might be advisable in clinically diagnosed AD patients (Van Mossevelde et al., 2016). In this study, the *TBK1*p.D534H carrier is a LOAD patient with a late‐onset dementia family history, indicating the pathogenicity of the *TBK1*p.D534H variant. However, 75% of *TBK1* mutation carriers developed the disease after the age of 62 years, while some remained unaffected at the age of >80 years (Van Mossevelde et al., 2016). Moreover, in silico analysis predicted probably damaging, which also supported that* TBK1*p.D534H was pathogenic; however, the underlying mechanism is yet to be investigated.

Nevertheless, the present study had several limitations. First, the *PSEN2*and *TBK1* mutations were not confirmed cosegregations in the families. Second, the study failed to demonstrate any changes in the biomarker in cerebrospinal fluid and during autopsy verification.

In summary, we identified five variants in a Han Chinese cohort. A novel, probably damaging FTLD gene *TBK1* variant with a typical AD phenotype was detected. Also, the phenotypic characteristics of *PSEN1* p.L226R, which was not reported previously, a *PSEN2* pathogenic mutation, and two likely benign *MAPT* variants were described. Hence, screening for mutations in other dementia genes might be advisable in clinically diagnosed AD patients.

## CONFLICT OF INTEREST

The authors have no actual or potential conflicts of interest.

## References

[brb31180-bib-0001] Bagyinszky, E. , Park, S. A. , Kim, H. J. , Choi, S. H. , An, S. S. , & Kim, S. Y. (2016). PSEN1 L226F mutation in a patient with early‐onset Alzheimer's disease in Korea. Clinical Interventions in Aging, 11, 1433–1440. 27785004 10.2147/CIA.S111821 PMC5066688

[brb31180-bib-0002] Bagyinszky, E. , Youn, Y. C. , An, S. S. , & Kim, S. (2016). Mutations, associated with early‐onset Alzheimer's disease, discovered in Asian countries. Clinical Interventions in Aging, 11, 1467–1488. 27799753 10.2147/CIA.S116218 PMC5074729

[brb31180-bib-0004] Brouwers, N. , Nuytemans, K. , van der Zee, J. , Gijselinck, I. , Engelborghs, S. , Theuns, J. , … Sleegers, K. (2007). Alzheimer and Parkinson diagnoses in progranulin null mutation carriers in an extended founder family. Archives of Neurology, 64(10), 1436–1446. 10.1001/archneur.64.10.1436 17923627

[brb31180-bib-0005] Cacace, R. , VanCauwenberghe, C. , Bettens, K. , Gijselinck, I. , van derZee, J. , Engelborghs, S. , … Sleegers, K. (2013). C9orf72 G4C2 repeat expansions in Alzheimer's disease and mild cognitive impairment. Neurobiology of Aging, 34(6), 1712.e1–1712.e7. 10.1016/j.neurobiolaging.2012.12.019 23352322

[brb31180-bib-0007] Che, X. Q. , Zhao, Q. H. , Huang, Y. , Li, X. , Ren, R. J. , Chen, S. D. , … Wang, G. (2018). Mutation screening of the CHCHD2 gene for Alzheimer's disease and frontotemporal dementia in Chinese mainland population. Journal of Alzheimer's Disease: JAD, 61(4), 1283–1288. 10.3233/JAD-170692 29376860

[brb31180-bib-0008] Coleman, P. , Kurlan, R. , Crook, R. , Werner, J. , & Hardy, J. (2004). A new presenilin Alzheimer's disease case confirms the helical alignment of pathogenic mutations in transmembrane domain 5. NeuroscienceLetters, 364(3), 139–140. 10.1016/j.neulet.2004.04.030 15196662

[brb31180-bib-0009] Deng, B. , Lian, Y. , Wang, X. , Zeng, F. , Jiao, B. , Wang, Y. R. , … Wang, Y. J. (2014). Identification of a novel mutation in the presenilin 1 gene in a Chinese Alzheimer's disease family. Neurotoxicity Research, 26(3), 211–215. 10.1007/s12640-014-9462-3 24737487

[brb31180-bib-0010] Dong, J. , Qin, W. , Wei, C. , Tang, Y. , Wang, Q. , & Jia, J. (2017). A novel PSEN1 K311R mutation discovered in Chinese families with late‐onset Alzheimer's disease affects amyloid‐beta production and tau phosphorylation. Journal of Alzheimer's Disease: JAD, 57(2), 613–623. 28269784 10.3233/JAD-161188

[brb31180-bib-0011] Freischmidt, A. , Muller, K. , Ludolph, A. C. , Weishaupt, J. H. , & Andersen, P. M. (2017). Association of mutations in TBK1 with sporadic and familial amyotrophic lateral sclerosis and frontotemporal dementia. JamaNeurology, 74(1), 110–113. 10.1001/jamaneurol.2016.3712 27892983

[brb31180-bib-0012] Freischmidt, A. , Wieland, T. , Richter, B. , Ruf, W. , Schaeffer, V. , Muller, K. , … Weishaupt, J. H. (2015). Haploinsufficiency of TBK1 causes familial ALS and fronto‐temporal dementia. NatureNeuroscience, 18(5), 631–636. 10.1038/nn.4000 25803835

[brb31180-bib-0013] Giri, M. , Zhang, M. , & Lu, Y. (2016). Genes associated with Alzheimer's disease: an overview and current status. Clinical Interventions in Aging, 11, 665–681. 27274215 10.2147/CIA.S105769 PMC4876682

[brb31180-bib-0014] Goedert, M. , & Spillantini, M. G. (2011). Pathogenesis of the tauopathies. Journal of Molecular Neuroscience: MN, 45(3), 425–431. 10.1007/s12031-011-9593-4 21785996

[brb31180-bib-0015] Gomez‐Tortosa, E. , Barquero, S. , Baron, M. , Gil‐Neciga, E. , Castellanos, F. , Zurdo, M. , … Jimenez‐Escrig, A. (2010). Clinical‐genetic correlations in familial Alzheimer's disease caused by presenilin 1 mutations. Journal of Alzheimer's Disease: JAD, 19(3), 873–884. 10.3233/JAD-2010-1292 20157243

[brb31180-bib-0016] He, Z. , Zhang, D. , Renton, A. E. , Li, B. , Zhao, L. , Wang, G. T. , … Leal, S. M. (2017). The rare‐variant generalized disequilibrium test for association analysis of nuclear and extended pedigrees with application to Alzheimer disease WGS data. American Journal of Human Genetics, 100(2), 193–204. 10.1016/j.ajhg.2016.12.001 28065470 PMC5294711

[brb31180-bib-0017] Hooli, B. V. , Kovacs‐Vajna, Z. M. , Mullin, K. , Blumenthal, M. A. , Mattheisen, M. , Zhang, C. , … Tanzi, R. E. (2014). Rare autosomal copy number variations in early‐onset familial Alzheimer's disease. Molecular Psychiatry, 19(6), 676–681. 10.1038/mp.2013.77 23752245

[brb31180-bib-0018] Jayadev, S. , Leverenz, J. B. , Steinbart, E. , Stahl, J. , Klunk, W. , Yu, C. E. , & Bird, T. D. (2010). Alzheimer's disease phenotypes and genotypes associated with mutations in presenilin 2. BrainA Journal of Neurology, 133(Pt 4), 1143–1154. 10.1093/brain/awq033 PMC2850581 20375137

[brb31180-bib-0019] Jiang, H. Y. , Li, G. D. , Dai, S. X. , Bi, R. , Zhang, D. F. , Li, Z. F. , … Yao, Y. G. (2015). Identification of PSEN1 mutations p. M233L and p.R352C in Han Chinese families with early‐onset familial Alzheimer's disease. Neurobiology of Aging, 36(3):1602.e3–1602.e6. 10.1016/j.neurobiolaging.2014.11.009 25595498

[brb31180-bib-0020] Landeiro, F. , Wace, H. , Ghinai, I. , Nye, E. , Mughal, S. , Walsh, K. , … ROADMAP Group (2018). Resource utilisation and costs in predementia and dementia: A systematic review protocol. British Medical Journal Open, 8(1), e019060 10.1136/bmjopen-2017-019060 PMC5988053 29362261

[brb31180-bib-0021] McKhann, G. , Drachman, D. , Folstein, M. , Katzman, R. , Price, D. , & Stadlan, E. M. (1984). Clinical diagnosis of Alzheimer's disease: Report of the NINCDS‐ADRDA Work Group under the auspices of Department of Health and Human Services Task Force on Alzheimer's Disease. Neurology, 34(7), 939–944. 10.1212/WNL.34.7.939 6610841

[brb31180-bib-0022] Mendez, M. F. (2017). Early‐onset Alzheimer disease. Neurologic Clinics, 35(2), 263–281. 10.1016/j.ncl.2017.01.005 28410659 PMC5407192

[brb31180-bib-0023] Park, S. A. , Ahn, S. I. , & Gallo, J. M. (2016). Tau mis‐splicing in the pathogenesis of neurodegenerative disorders. BMB Reports, 49(8), 405–413. 10.5483/BMBRep.2016.49.8.084 27222125 PMC5070727

[brb31180-bib-0024] Piccoli, E. , Rossi, G. , Rossi, T. , Pelliccioni, G. , D'Amato, I. , Tagliavini, F. , & Di Fede, G. (2016). Novel PSEN1 mutations (H214N and R220P) associated with familial Alzheimer's disease identified by targeted exome sequencing. Neurobiology of Aging, 40, 192.e7–192.e11. 10.1016/j.neurobiolaging.2016.01.134 26925509

[brb31180-bib-0025] Pottier, C. , Ravenscroft, T. A. , Brown, P. H. , Finch, N. A. , Baker, M. , Parsons, M. , … Rademakers, R. (2016). TYROBP genetic variants in early‐onset Alzheimer's disease. Neurobiology of Aging, 48, 222.e9–222.e15. 10.1016/j.neurobiolaging.2016.07.028 PMC5159294 27658901

[brb31180-bib-0026] Rademakers, R. , Cruts, M. , & van Broeckhoven, C. (2004). The role of tau (MAPT) in frontotemporal dementia and related tauopathies. Human Mutation, 24(4), 277–295. 10.1002/humu.20086 15365985

[brb31180-bib-0027] Rademakers, R. , Dermaut, B. , Peeters, K. , Cruts, M. , Heutink, P. , Goate, A. , & Van Broeckhoven, C. (2003). Tau (MAPT) mutation Arg406Trp presenting clinically with Alzheimer disease does not share a common founder in Western Europe. Human Mutation, 22(5), 409–411. 10.1002/humu.10269 14517953

[brb31180-bib-0028] Sergeant, N. , Delacourte, A. , & Buee, L. (2005). Tau protein as a differential biomarker of tauopathies. Biochimica Et Biophysica Acta, 1739(2–3), 179–197. 10.1016/j.bbadis.2004.06.020 15615637

[brb31180-bib-0029] Shea, Y. F. , Chu, L. W. , Chan, A. O. , Ha, J. , Li, Y. , & Song, Y. Q. (2016). A systematic review of familial Alzheimer's disease: Differences in presentation of clinical features among three mutated genes and potential ethnic differences. Journal of the Formosan Medical Association, 115(2), 67–75. 10.1016/j.jfma.2015.08.004 26337232

[brb31180-bib-0030] Shi, Z. , Wang, Y. , Liu, S. , Liu, M. , Liu, S. , Zhou, Y. , … Ji, Y. (2015). Clinical and neuroimaging characterization of Chinese dementia patients with PSEN1 and PSEN2 mutations. Dementia and Geriatric Cognitive Disorders, 39(1–2), 32–40. 25323700 10.1159/000366272

[brb31180-bib-0031] Sutovsky, S. , Smolek, T. , Turcani, P. , Petrovic, R. , Brandoburova, P. , Jadhav, S. , … Zilka, N. (2018). Neuropathology and biochemistry of early onset familial Alzheimer's disease caused by presenilin‐1 missense mutation Thr116Asn. Journal of Neural Transmission, 125(6), 965–976. 10.1007/s00702-018-1850-z 29404783

[brb31180-bib-0032] van der Zee, J. , Gijselinck, I. , Van Mossevelde, S. , Perrone, F. , Dillen, L. , Heeman, B. , … Testi , S. (2017). TBK1 mutation spectrum in an extended European patient Cohort with frontotemporal dementia and amyotrophic lateral sclerosis. Human Mutation, 38(3), 297–309. 28008748 10.1002/humu.23161 PMC5324646

[brb31180-bib-0033] Van Mossevelde, S. , van der Zee, J. , Gijselinck, I. , Engelborghs, S. , Sieben, A. , Van Langenhove, T. , … Belgian Neurology Consortium (2016). Clinical features of TBK1 carriers compared with C9orf72, GRN and non‐mutation carriers in a Belgian cohort. BrainA Journal of Neurology, 139(Pt 2), 452–467. 10.1093/brain/awv358 PMC4805085 26674655

[brb31180-bib-0034] Vardarajan, B. N. , Ghani, M. , Kahn, A. , Sheikh, S. , Sato, C. , Barral, S. , … Mayeux, R. (2015). Rare coding mutations identified by sequencing of Alzheimer disease genome‐wide association studies loci. Annals of Neurology, 78(3), 487–498. 10.1002/ana.24466 26101835 PMC4546546

[brb31180-bib-0035] Verheijen, J. , van derZee, J. , Gijselinck, I. , Van denBossche, T. , Dillen, L. , Heeman, B. … EU EOD Consortium (2018). Common and rare TBK1 variants in early‐onset Alzheimer disease in a European cohort. Neurobiology of Aging, 62, 245.e1–245.e7. 10.1016/j.neurobiolaging.2017.10.012 29146049

[brb31180-bib-0036] Xia, M. , Chen, S. , Shi, Y. , Huang, Y. , Xu, J. , Zhao, T. , …. Zhang, J. (2015). Probable novel PSEN2 Pro123Leu mutation in a Chinese Han family of Alzheimer's disease. Neurobiology of Aging, 36(12), 3334.e13–3334.e18. 10.1016/j.neurobiolaging.2015.09.003 26422362

[brb31180-bib-0037] Yagi, R. , Miyamoto, R. , Morino, H. , Izumi, Y. , Kuramochi, M. , Kurashige, T. , … Kawakami, H. (2014). Detecting gene mutations in Japanese Alzheimer's patients by semiconductor sequencing. Neurobiology of Aging, 35(7), 1780.e1–1780.e5. 10.1016/j.neurobiolaging.2014.01.023 24559647

[brb31180-bib-0038] Zekanowski, C. , Golan, M. P. , Krzysko, K. A. , Lipczynska‐Lojkowska, W. , Filipek, S. , Kowalska, A. , … Kuznicki, J. (2006). Two novel presenilin 1 gene mutations connected with frontotemporal dementia‐like clinical phenotype: Genetic and bioinformatic assessment. Experimental Neurology, 200(1), 82–88. 10.1016/j.expneurol.2006.01.022 16546171

[brb31180-bib-0039] Zhan, Y. , Zheng, H. , Wang, C. , Rong, Z. , Xiao, N. , Ma, Q. , & Zhang, Y. W. (2017). A novel presenilin 1 mutation (F388L) identified in a Chinese family with early‐onset Alzheimer's disease. Neurobiology of Aging, 50, 168.e1–168.e4. 10.1016/j.neurobiolaging.2016.10.010 27836335

