# Supplementary material for: Gene mutations in a Han Chinese Alzheimer's disease cohort
Source: Brain Behav. 2018 Dec 14;9(1):e01180. doi: 10.1002/brb3.1180 (PMC6346667; doi:10.1002/brb3.1180)
Supplement: Supplementary file 1 [file BRB3-9-e01180-s001.docx]

| A2M | ATN1 | CR1 | HNRNPA1 | MPO | PLD3 | SNCB | TRPM7 |
| --- | --- | --- | --- | --- | --- | --- | --- |
| ACE | ATP13A2 | DNMT1 | HNRNPA2B1 | NOS3 | PRNP | SORL1 | TYROBP |
| ACHE | BCHE | FAM134B | ITM2B | NOTCH3 | PSEN1 | SQSTM1 | UBQLN2 |
| ADAM10 | C9orf72 | GBA | KLK1 | NPC1 | PSEN2 | TARDBP | VCP |
| APBB2 | CHMP2B | GRN | LOC643387 | PAXIP1 | RPS27A | TBK1 |  |
| APOE | CHRM1 | GSTO1 | MAPT | PICALM | SLC6A4 | TNFSF14 |  |
| APP | CLU | HFE | MEOX2 | PLAU | SNCA | TREM2 |  |
