# Supplementary material for: Gene mutations in a Han Chinese Alzheimer's disease cohort
Source: Brain Behav. 2018 Dec 14;9(1):e01180. doi: 10.1002/brb3.1180 (PMC6346667; doi:10.1002/brb3.1180)
Supplement: Supplementary file 2 [file BRB3-9-e01180-s002.doc]

| gene | Transcript number | Nucleotide change | Amino acid change |
| --- | --- | --- | --- |
| ACE | NM_000789 | c.1057G>A | p.D353N |
| ACE | NM_000789 | c.1910C>T | p.P637L |
| ACEH | NM_015831 | c.806C>T | p.T269M |
| ATP13A2 | NM_022089 | c.1065C>T | splicing |
| BCHE | NM_000055 | c.1628G>A | p.R543H |
| NPC1 | NM_000271 | c.3527C>T | p.T1176M |
| DNMT1 | NM_001130823 | c.1064G>A | p.R355H |
| TREM2 | NM_018965 | c.574G>A | p.A192T |
| PLAU | NM_002658 | c.562G>A | p.E188K |
| TYROBP | NM_003332 | c.67C>T | p.R23C |
